# Effect of Potassium Permanganate Modification on Plasticized Spinning Polyacrylonitrile Fibers with Different Diameters

**DOI:** 10.3390/polym10121330

**Published:** 2018-12-01

**Authors:** Xiang Li, Xiaonan Dang

**Affiliations:** 1 School of medicine and chemical engineering, Zhenjiang College, Zhenjiang 212003, China; 2 Zhenjiang Key Laboratory of Functional Chemistry, Zhenjiang 212003, China; 3 Zhenjiang Customs, Zhenjiang 212003, China; dangxn@jsciq.gov.cn

**Keywords:** plasticized spinning, diameter, potassium permanganate, chemical modification

## Abstract

Plasticized spinning polyacrylonitrile (PAN) fibers with different diameters were chemically modified by potassium permanganate (KMnO_4_). The modification effects of different diameter fibers were studied for the first time. Differential scanning calorimetry (DSC) results show that, compared with the large diameter ones, small diameter modified fibers show lower cyclization starting temperature (*T_i_*) and activation energy (E). Both kinds of fibers exhibit better modification effects compared with solution-spun fibers. For the small diameter fibers, chemical modification can occur at low treatment temperature, even at 70 °C. X-ray diffraction analysis (XRD) results show that modification not only occurs in the amorphous region of the fibers but also in the crystalline region.

## 1. Introduction

Plasticized spinning is a novel method for preparing polyacrylonitrile fibers. This method is very attractive because it requires only a small amount of plasticizer and the fiber structure is controllable [1]. Currently, an ionic liquid possesses characteristics of strong polarity and high-temperature stability [2] and has exhibited significant advantages over other traditional plasticizers such as ethylene carbonate and propylene carbonate. 1-butyl-3-methylimidazolium chloride, as a water-soluble ionic liquid [3], is the most suitable plasticizer for PAN to achieve plasticized spinning [4]. Nevertheless, high-performance plasticized spinning PAN fibers still cannot be easily fabricated since the fiber properties are affected by many process parameters during the spinning process. Recently, a lot of effort has been made to prepare PAN fibers with excellent properties. [5,6] Some properties of the prepared fibers, such as tensile strength and initial modulus, have been comparable to those of commercial PAN fibers. However, until now, the relevant research has still been focused on improving the performance of the precursors [5,6,7]. The subsequent modification, pre-oxidation and carbonization processes are rarely reported. 

Modification is carried out on the PAN fibers following the spinning process in order to reduce the activation energy of cyclization [8], decrease the stabilization exotherm [9], and increase the speed of the cyclization reaction [10]. It ultimately improves the performance of the carbon fibers. Chemical modification involves the impregnation of the fibers with certain chemicals under various conditions [11]. One good example of a chemical treatment is the impregnation of the fibers with potassium permanganate (KMnO_4_) [12]. It is foreseeable that, at a modification temperature of 85 °C, the KMnO_4_ modification effect is remarkable for those PAN fibers fabricated by solution spinning [8]. Interestingly, for plasticized spinning PAN fibers, oxygen-containing groups are generated during the spinning process, which also can reduce the cyclization reaction time [13]. If those fibers are further modified by KMnO_4_, a good modification effect can be obtained even at a lower treatment temperature.

Based on our previous study [14], the entire spinning process is controllable as the final fiber diameter is 16.6 μm. In addition, the fibers can reach the optimum mechanical properties. Until now, fibers with a diameter of 10.2 μm have been successfully fabricated in our laboratory, although the mechanical properties are not satisfactory and are induced by the instability of the spinning process. It is still desirable to study the influence of the fiber diameter on the modification effect because it will provide us with important information to further improve the fiber structure.

## 2.Material and Methods

### 2.1. Materials

**Figure 1:**
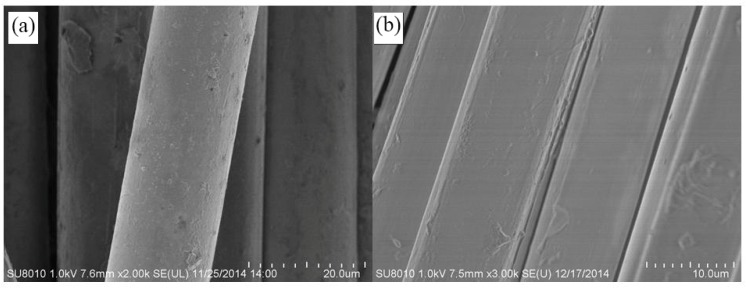
Apparent morphology of the fibers. (**a**) Large diameter fibers; (**b**) small diameter fibers.

PAN powder (*M*_w_ = 7.2 × 10^4^ g/mol, *M*_n_ = 3.3 × 10^4^ g/mol, *M*_w_/*M*_n_ = 2.22) was purchased from Sinopharm Chemical Reagent Co., Ltd. (Shanghai, China) After the plasticized spinning process, the PAN fibers with diameters of 16.6 μm and 10.2 μm were prepared. The mechanical properties of different diameter fibers are shown in Table 1. The apparent morphology of the fibers can be seen in Figure 1. 

### 2.2. Preparation of Samples

KMnO_4_ was purchased from the Shanghai Chemical Reagents Co., Ltd. (Shanghai, China).

Preparation of samples: the fibers were soaked in 8 wt % KMnO_4_ solution at 70 °C, 75 °C and 80 °C for 10 min. Then the fibers were washed thoroughly with distilled water and dried at 70 °C for 2 h.

### 2.3. Characterization

Differential scanning calorimetry (Q20, Delaware, TA, USA) was used to analyze the thermal performance of the fibers at a heating rate of 10 °C/min under a nitrogen atmosphere. The fibers were cut as short as possible and 3 g of the fibers was accurately weighed for each measurement. 

X-ray diffraction analysis (XRD) using CuKα (L = 0.1542 nm) was conducted using an X-ray diffractometer (D/Max-2550 PC, RIGAKU, Tokyo, Japan). The operating voltage and electricity were 30 kV and 10 mA respectively. The average crystal size (*L_c_*) was calculated according to the Scherrer equation [15].

(1) $$L_{C}=\frac{k\lambda }{\beta \cos\theta }$$

where *k* is the apparatus constant, which was taken to be 0.89; *λ* = 0.1542 nm is the wavelength of the X-rays; *β* is the full width at half maximum in radians at 2*θ* = 17°.

The crystallinity was determined from the XRD patterns of the fibers according to the formula calculated by Hinrichsen method [16].

(2) $$C\%=\frac{Ac}{Ac+Aa}$$

where *Ac* is the integral area of the crystalline region, and *Aa* is the integral area of the amorphous region.

A sound velocity orientation tester (SCY-III, Donghua University, Shanghai, China) was adopted to determine the sound velocity orientation degree of the fibers [6]. 

## 3. Results 

**Figure 2:**
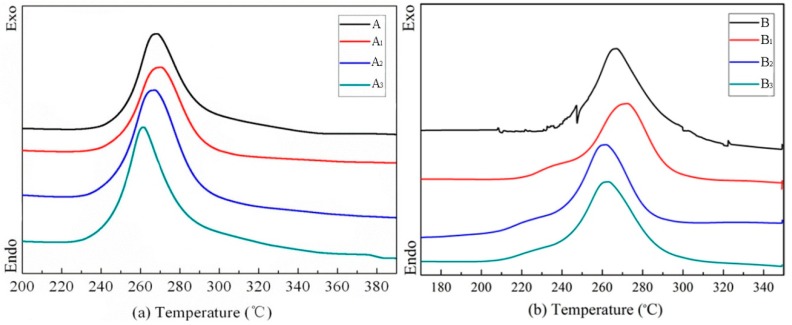
DSC curves of different diameter PAN fibers after modification. (**a**) Large diameter; (**b**) small diameter.

Table 2 and Figure 2 show the DSC data and DSC curves of PAN fibers with different diameters after modification. A and B samples in Table 2 shows the same cyclization starting temperature (*T_i_*) and cyclization temperature (*T*). However, the heat released (ΔH) of B sample is significantly higher than that of A sample. The *T_i_* of B sample is obviously lower than that of A sample under the same treatment temperature, indicating that the catalytic modification effect is more remarkable for small diameter fibers. A lower *T_i_* for small diameter fibers means that the initiation of the cyclization reaction becomes easier in the subsequent stabilization stage, which is beneficial to improve the speed of the stabilization.

For large diameter PAN fibers (16.6 μm), *T_i_* hardly changed at 70 °C and the heat released (ΔH) was reduced from 403 to 335.2 J g^−1^. This suggests that modification does not occur at this temperature and the presence of KMnO_4_ on the fibers even hinders the cyclization reaction. With an increasing treatment temperature, the *T_i_* decreased and ΔH increased, meanwhile, the exothermic range (Δ*T*) increased as well. This indicates that the cyclization process of the fibers becomes moderate and more C≡N groups are involved in cyclization reaction with an increase in temperature. 

For small diameter PAN fibers (10.2 μm), the *T_i_* obviously reduced from 229.8 to 213.6 °C at 70 °C, suggesting that the small diameter fibers exhibit a better modification effect. Meanwhile, the ΔH decreased with an increasing heat treatment temperature; this may be due to the increased number of C≡N groups that have been converted to conjugated C=N groups during the catalytic modification process.

**Figure 3:**
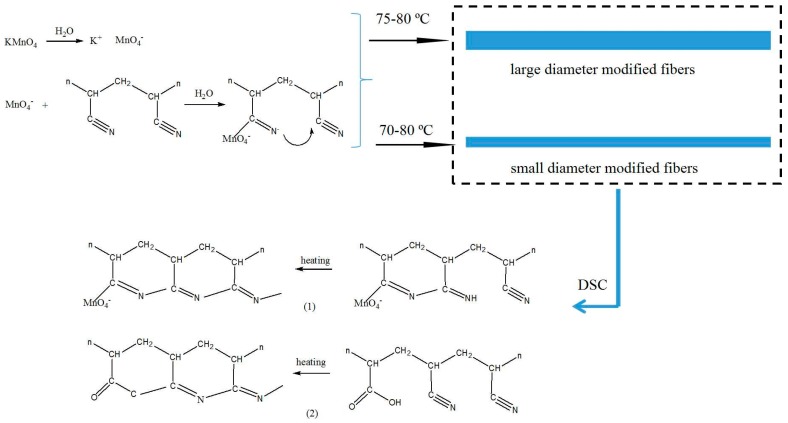
The possible cyclization process of the fibers. (**1**) The cyclization reaction initiated by C=NR (R may be H) groups formed during the modification process; (**2**) the cyclization reaction initiated by oxygen groups generated during the plasticized spinning process.

Therefore, the possible cyclization process of plasticized spinning fibers can be observed in Figure 3. The cyclization reaction can be initiated by –C=NR (R may be H) groups formed during the modification process. These groups have been confirmed by infrared spectra in our previous study [17]. Simultaneously, the reaction also can be excited by the oxygen groups (e.g., –COOH groups) generated during the plasticized spinning process [18].

The DSC peak positions with different heating rates are summarized in Table 3. The activation energy can be obtained by fitting Kissinger [19].

(3) $$-\frac{E}{R}=\frac{d\ln(\Phi /T_{p}{}^{2})}{d(1/T_{p})}$$

This formula can also be written as:

(4) $$\ln\frac{\Phi }{T_{p}{}^{2}}=-\frac{E}{R}\frac{1}{T_{P}}+\ln\frac{RA}{E}$$

where *E* is the activation energy, Φ is the heating rate (°C/min), *R* is molar gas constant (8.314 J·mol^−1^·K^−1^), *A* is the frequency factor, and *T_P_* is the peak temperature (in Kelvin).

**Figure 4:**
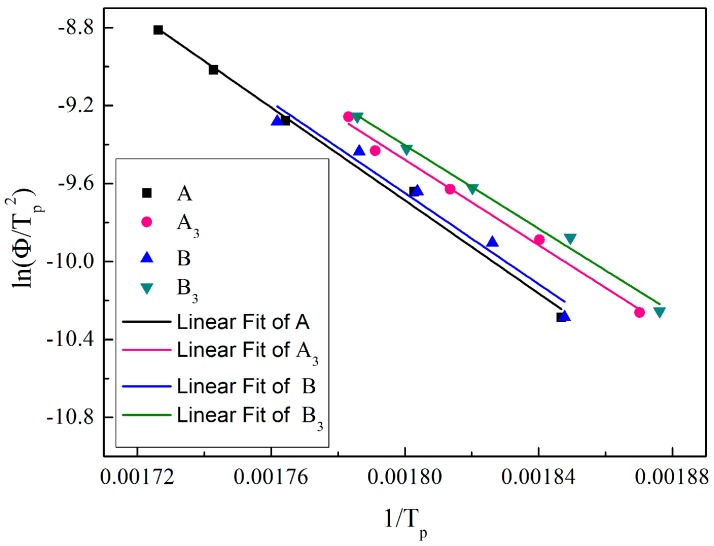
lnΦ/(*T_p_*)^2^ as a function of (*T_p_*)^−1^.

The Kissinger plots are shown in Figure 4, $-\frac{E}{R}$ is the slope of the fitted curve, thus *E* can be calculated. Further, *A* can also be calculated by the intercept of the Y coordinate. Thus, the activation energies for A, A_3_, B and B_3_ are 99.15 kJ/mol, 90.96 kJ/mol, 96.96 kJ/mol and 88.98 kJ/mol respectively. 

It can be intuitively seen that small diameter fibers possess a relatively lower *E* compared with large diameter ones. Meanwhile, both A_3_ and B_3_ show a lower *E* value compared with solution-spun PAN fibers modified at a higher temperature of 85 °C [10,12]. In our previous study [17], it is believed that a special ladder structure has been formed during the plasticized spinning. A lower *E* value for plasticized spinning fibers reveals the structure superiority, although this structure has not been studied in depth. 

**Figure 5:**
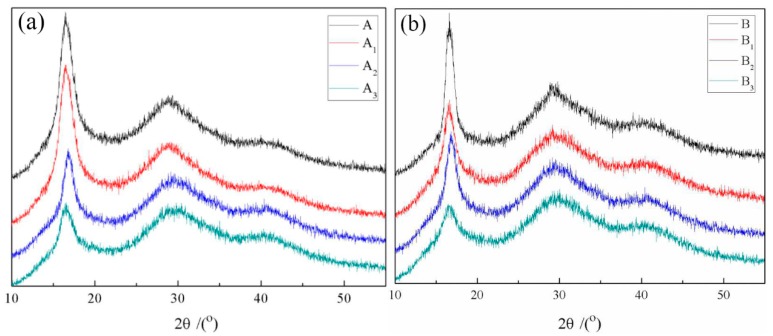
XRD curves of different diameter PAN fibers after modification. (**a**) XRD curves of large diameter; (**b**) XRD curves of small diameter.

Table 4 and Figure 5 show the XRD data and curves of PAN fibers after modification. It can be observed that modification occurs not only in the amorphous region of the fibers but also in the crystalline region, resulting in a decrease in crystallinity. For small diameter PAN fibers, the crystallinity, crystal size and orientation degree are obviously reduced with an increasing treatment temperature. The better modification effect for small diameter fibers may be due to the larger surface area at the same quality, which may introduce more MnO_4_^−^ active sites. For large diameter PAN fibers, the crystallinity, crystal size and orientation degree were almost unchanged as treated at 70 °C, this also suggests that modification hardly take place at this temperature.

## 4. Conclusions

For plasticized spinning fibers, two kinds of fibers show a good modification effect. However, the modification effect is more remarkable for small diameter fibers. Modification not only occurs in the amorphous region but also in the crystalline region. For large diameter fibers, modification hardly occurs at 70 °C.

## Figures and Tables

**Table 1:** Mechanical properties of the plasticized spinning PAN fibers.

Fiber Type	Fiber Fineness/dtex	Fiber Diameter/μm	Elongation at Break/%	Breaking Strength/(cN/dtex)	Initial Modulus/(cN/dtex)
Large diameter	2.33	16.6	9.26	5.95	100.92
Small diameter	1.16	10.2	7.58	5	83.56

**Table 2:** DSC data of PAN fibers with different diameters after modification.

Diameter/μm	Temperature/°C	Abbreviation	*T_i_*/°C	*T_f_*/°C	Δ*T*	*T*/°C	ΔH/J g^−1^
16.6	-	A	229.5	353.2	123.72	268.52	403
	70	A_1_	229.8	364.17	139.39	270.43	335.2
	75	A_2_	226.1	371.79	145.67	267.39	454.5
	80	A_3_	224	382.81	158.82	261.69	480.5
10.2	-	B	229.8	346.8	117	268.23	451.2
	70	B_1_	213.6	325.84	112.23	272.39	411.8
	75	B_2_	207.5	313.82	106.31	261.52	404.4
	80	B_3_	199	312.37	113.37	260	400.6

Note: *T_f_* means the cyclization finishing temperature.

**Table 3:** DSC data of plasticized spinning PAN fibers.

Heating Rate/(°C/min)	16.6 μm		10.2 μm	
	A	A_3_	B	B_3_
10	268.5	261.7	268.2	260
15	281.7	270.4	274.6	267.7
20	293.8	278.4	281.4	276.4
25	300.8	285.3	286.8	282.4
30	306.3	287.8	294.6	287

**Table 4:** XRD data of different diameter PAN fibers after modification.

Fiber Type	Crystallinity/%	Crystal Size/nm	Orientation Degree/%
A	67.4	4.71	85.3
A_1_	67.1	4.68	85.5
A_2_	63.04	4.45	79.6
A_3_	59.67	4.25	75.4
B	66.6	4.63	83.4
B_1_	62.15	4.14	74.5
B_2_	56.52	3.98	67.1
B_3_	53.12	3.46	62.7

## References

[1] Li X., Qin A.W., Zhao X.Z., Liu D.P., Wang H.Y., He C.J. (2015). Drawing dependent structures, mechanical properties and cyclization behaviors of polyacrylonitrile and polyacrylonitrile/carbon nanotube composite fibers prepared by plasticized spinning. Phys. Chem. Chem. Phys..

[2] Matandabuzo M., Ajibade P.A. (2018). Synthesis and surface functionalization of multi-walled carbon nanotubes with imidazolium and pyridinium-based ionic liquids: Thermal stability, dispersibility and hydrophobicity characteristics. J. Mol. Lid..

[3] Hiraga Y., Koyama K., Sato Y., Smith R.L. (2017). High pressure densities for mixed ionic liquids having different functionalities: 1-butyl-3-methylimidazolium chloride and 1-butyl-3-methylimidazolium bis(trifluoromethylsulfonyl)imide. J. Chem. Thermodyn..

[4] Li X., Qin A.W., Zhao X.Z., He C.J. (2015). The plasticized spinning and cyclization behaviors of polyacrylonitrile/functionalized carbon nanotube (PAN/CNT) fibers. RSC Adv..

[5] Liu S.P., Han K.Q., Chen L., Zheng Y., Yu M.H., Li J.Q., Yang Z. (2015). Influence of External Tension on the Structure and Properties of Melt-Spun PAN Precursor Fibers during Thermal Oxidation. Macromol. Mater. Eng..

[6] Liu S.P., Han K.Q., Chen L., Zheng Y., Yu M.H. (2015). Structure and properties of partially cyclized polyacrylonitrile-based carbon fiber—Precursor fiber prepared by melt-spun with ionic liquid as the medium of processing. Polym. Eng. Sci..

[7] Liu S.P., Han K.Q., Chen L., Zheng Y., Yu M.H. (2015). Influence of air circulation on the structure and properties of melt-spun PAN precursor fibers during thermal oxidation. RSC Adv..

[8] Mittal J., Mathur R.B., Bahl O.P., Inagaki M. (1998). Post spinning treatment of PAN fibers using succinic acid to produce high performance carbon fibers. Carbon.

[9] Mathur R.B., Mittal J., Bahl O.P., Sandle N.K. (1994). Characteristics of KMnO_4_-modified PAN fibres—Its influence on the resulting carbon fibres’ properties. Carbon.

[10] Bahl D.P., Mathur R.B., Dhami T.L. (1985). Modification of polyacrylonitrile fibres to make them suitable for conversion into high performance carbon fibres. Mater. Sci. Eng..

[11] Zhang W.X., Wang Y.Z. (2002). Manufacture of carbon fibers from polyacrylonitrile precursors treated with CoSO_4_. J. Appl. Polym. Sci..

[12] Yusof N., Ismail A.F. (2002). Post spinning and pyrolysis processes of polyacrylonitrile (PAN)-based carbon fiber and activated carbon fiber: A review. J. Anal. Appl. Pyrol..

[13] Tian Y.C., Han K.G., Zhang W.H., Zhang J.J., Rong H.P., Wang D., Yan B., Liu S.P., Yu M.H. (2003). Influence of residence time on the structure of polyacrylonitrile in ionic liquids during melt spinning process. Mater. Lett..

[14] Li X., Li Z.L., Dang X.N., Luan D., Wang F., Chen H.Y., Wang C.T. (2017). Structural and thermal property changes of plasticized spinning polyacrylonitrile fibers under different spinning speeds. J. Appl. Polym. Sci..

[15] Gupta V.B., Kumar S. (1981). The effect of heat setting on the structure and mechanical properties of poly(ethylene terephthalate) fiber. I. Structural changes. J. Appl. Polym. Sci..

[16] Ju A.Q., Guang S.Y., Xu H.Y. (2013). Effect of comonomer structure on the stabilization and spinnability of polyacrylonitrile copolymers. Carbon.

[17] Li X., Qin A.W., Zhao X.Z., Ma B.M., He C.J. (2014). The plasticization mechanism of polyacrylonitrile/1-butyl-3-methylimidazolium chloride system. Polymer.

[18] Li X., Liu C.Y., Qin A.W., Zhao X.Z., He C.J. (2013). Effect of processing conditions on properties of KMnO_4_-treated PAN fibers. Adv. Mater. Res..

[19] Morris E.A., Weisenberger M.C., Abdallah M.G., Vautard F., Grappe H., Ozcan S., Paulauskas F.L., Eberle C., Jackson D., Mecham S.J. (2016). High performance carbon fibers from very high molecular weight polyacrylonitrile precursors. Carbon.

